# Uterine electromyography as a new predictor of extremely preterm birth: a multifactorial model integrating clinical and bioelectrical parameters

**DOI:** 10.1186/s12884-025-08539-3

**Published:** 2025-12-26

**Authors:** Jing Tang, Tianyuan Qi, Feiyan Li, Haiyan Lin, Xiaohui Ji, Xiaoyan Wang, Jianmei Lai, Chunwei Cao, Liqiong Zhu, Shuai Fu, Yan Yu, Shiyu Bai, Jianping Zhang, Qingxue Zhang, Yihong Guo, Hui Chen

**Affiliations:** 1 Reproductive Medicine Center, Sun Yat-Sen Memorial Hospital, Sun Yat-Sen University, No. 107 Yanjiang West Road, Guangzhou, 510120 People’s Republic of China; 2 Obstetrics and Gynecology department, Dongguan Maternal and Children Health Hospital, Dongguan, China; 3 Guangdong Provincial Clinical Research Center for Obstetrical and Gynecological Diseases, Guangzhou, China; 4 Obstetrics department, Sun Yat-Sen Memorial Hospital, Sun Yat-Sen University, Guangzhou, People’s Republic of China; 5 Obstetrics and Gynecology department, Shenzhen Baoan Women’s and Children’s Hospital, Shenzhen, China

**Keywords:** Electromyography, Extremely preterm infants, Predictive learning models

## Abstract

**Background:**

Extremely preterm birth (EPB), defined as delivery before 28 weeks of gestation, is a major contributor to neonatal morbidity and mortality. Accurate prediction of EPB is crucial for enabling timely interventions to improve neonatal outcomes and optimize resource allocation. Uterine electromyography (uEMG) is a non-invasive method that quantifies uterine electrical activity, offering potential for early EPB risk stratification. This study investigates the predictive value of uEMG parameters combined with traditional clinical risk factors for EPB.

**Methods:**

In this retrospective study, 276 singleton pregnant women underwent uEMG monitoring between 20+0 and 27+6 weeks of gestation at Sun Yat-sen Memorial Hospital (Guangzhou, China) from January 2018 to May 2025 were collected. The association of uEMG parameters (contraction frequency, average peak contraction intensity, and average contraction duration) with EPB were analyzed using logistic regression.Two predictive models were developed: a traditional model, including: assisted reproductive technology (ART), prior deliveries between 12 and 28 weeks, and transvaginal cervical length (TVCL); an enhanced model incorporating uEMG parameters (contraction frequency, average contraction duration) and clinical risk factor. The area under the receiver operating characteristic curve (AUC-ROC), precision-recall curve, calibration curve and decision curve analysis were used to assess predictive performance.

**Result:**

In total, 37 of 276 women (13.4%) experienced EPB, corresponding to 1 of 103 women in the no uterine contraction subgroup and 36 of 173 women in the uterine contraction subgroup. For model development, we restricted the analysis to the 173 women with detectable uterine contractions. Compared with the non-EPB group, the EPB group showed significantly higher contraction frequency, average peak contraction intensity, and average contraction duration. In multivariable analysis, higher contraction frequency and longer average contraction duration, ART, prior deliveries between 12 and 28 weeks, and shorter TVCL were independently associated with EPB. The uEMG model showed better discrimination than the traditional model (AUC-ROC 0.859, 95% CI 0.798–0.920 vs. 0.716, 95% CI 0.606–0.827; *P* < 0.05, DeLong test). In the derived nomogram, high-risk patients (score > 76) had markedly higher EPB rates than low-risk patients (training set: 42.9% vs. 10.0%; validation set: 55.6% vs. 0%; *P* < 0.001).

**Conclusion:**

uEMG parameters, particularly contraction frequency and average contraction duration, are independent predictors of EPB. A prediction model integrating these parameters with ART history, prior deliveries between 12 and 28 weeks, and TVCL provides good discrimination and clinical utility for EPB risk stratification. As a non-invasive and dynamic monitoring tool, uEMG may complement traditional assessment; however, our findings are derived from a single-center retrospective cohort and should be validated in larger, multicenter prospective studies before routine clinical implementation.

**Supplementary Information:**

The online version contains supplementary material available at 10.1186/s12884-025-08539-3.

## Introduction

Extremely preterm birth (EPB), defined as delivery before 28 weeks of gestation, affects approximately 2–5 per 1,000 pregnancies and remains one of the most challenging issues in maternal–fetal medicine [1]. Infants born at this gestational age are at exceptionally high risk of respiratory distress syndrome, severe neonatal sepsis, intraventricular hemorrhage, and long-term neurodevelopmental impairment, despite substantial advances in neonatal intensive care [2]. Improving the early identification of women at high risk of EPB is therefore critical to optimizing perinatal management, counseling, and resource allocation.

Preterm birth is driven by multifactorial etiologies, encompassing infectious, anatomical, placental abnormalities, uterine overdistension or stress and other immunologically problems [3, 4]. Among these mechanisms, abnormal uterine contractility is a central pathological mechanism contributing to preterm labor. Current strategies for risk stratification rely largely on clinical history and structural assessment. Established predictors include a history of preterm delivery, use of assisted reproductive technology (ART), and shortened transvaginal cervical length (TVCL). These factors are incorporated into guideline-recommended assessment algorithms and have improved the identification of women at risk of preterm birth [5]. However, they primarily reflect static anatomical or historical information and do not directly capture real-time uterine contractile activity. Therefore, exploring real-time, non-invasive technology to monitor uterine contractile activity holds significant clinical value for the early prediction and intervention of EPB.

Uterine electromyography (uEMG) is a non-invasive technique that records electrical activity of the uterine myometrium via surface electrodes, enabling quantitatively analysis of core parameters of uterine contractions [6–8]. Previous studies have reported that uEMG could detect uterine electrical activity and subtle contractions not readily captured by manual palpation or tocodynamometry, provide standardized electrical signal analysis to reduce subjective errors [9, 10]. Also, studies have shown that uEMG could effectively predict the risk of preterm birth at 37 weeks and outperform conventional tools in some settings [11–15]. However, most available data focus on overall preterm birth, and the predictive value of uEMG specifically for EPB has not been systematically evaluated.

To address this knowledge gap, we conducted a retrospective study of women undergoing uEMG monitoring between 20+0 and 27+6 weeks of gestation. Our primary aims were: (1) to assess the association between uEMG parameters (contraction frequency, average peak contraction intensity, and average contraction duration) and EPB; and (2) to develop and internally validate a prediction model that integrates uEMG parameters with established clinical risk factors, including ART, prior deliveries between 12–28 weeks, and TVCL. We hypothesized that uEMG-derived measures of uterine contractility would provide incremental predictive value over traditional clinical factors and enable more accurate risk stratification for EPB.

## Methods

### Study design and participants

This single-center retrospective study was conducted at Sun Yat-sen Memorial Hospital, Sun Yat-sen University, Guangzhou, China. We included women with singleton pregnancies who underwent uEMG monitoring between January 2018 and May 2025 and had documented delivery outcome.

This study was conducted in accordance with the Declaration of Helsinki. The protocol was reviewed and approved by the Medical Ethics Committee of Sun Yat-sen Memorial Hospital, Sun Yat-sen University (Approval No. SYSKY-2025-546-01). Given the retrospective design and use of de-identified data, the requirement for informed consent was waived by the Medical Ethics Committee of Sun Yat-sen Memorial Hospital, Sun Yat-sen University.

Inclusion criteria were: (1) Underwent uEMG monitoring during hospitalization; (2) clinical manifestations of vaginal bleeding with or without intermittent lower abdominal pain (3) Gestational age at uEMG monitoring between 20+0 and 27+6 weeks; (4) Singleton pregnancy. Exclusion criteria included: (1) congenital uterine malformation; (2) Termination of pregnancy due to severe complications, such as severe preeclampsia, massive antepartum hemorrhage, or other serious medical conditions; (3) Participants with systemic infection.

### Clinical management and acquisition of uterine electromyography data

Clinical management at our tertiary center followed standard operating procedures (SOPs) for women hospitalized with high risk of EPB. Admission and monitoring: admission between 20+0 and 27+6 weeks for vaginal bleeding with or without intermittent lower abdominal pain, baseline assessments (vital signs, infection screen, ultrasound, TVCL) and baseline uEMG obtained immediately upon admission. Treatment: initiation of tocolysis (e.g., atosiban) when regular contractions were present and no contraindications; antenatal corticosteroids for pregnancies at risk of delivery < 34 weeks; magnesium sulphate for fetal neuroprotection if delivery < 32 weeks was considered; antibiotics only when infection was suspected/confirmed; and consideration of cerclage for women with painless cervical dilation without contractions according to guideline-based criteria.

**Fig. 1:**
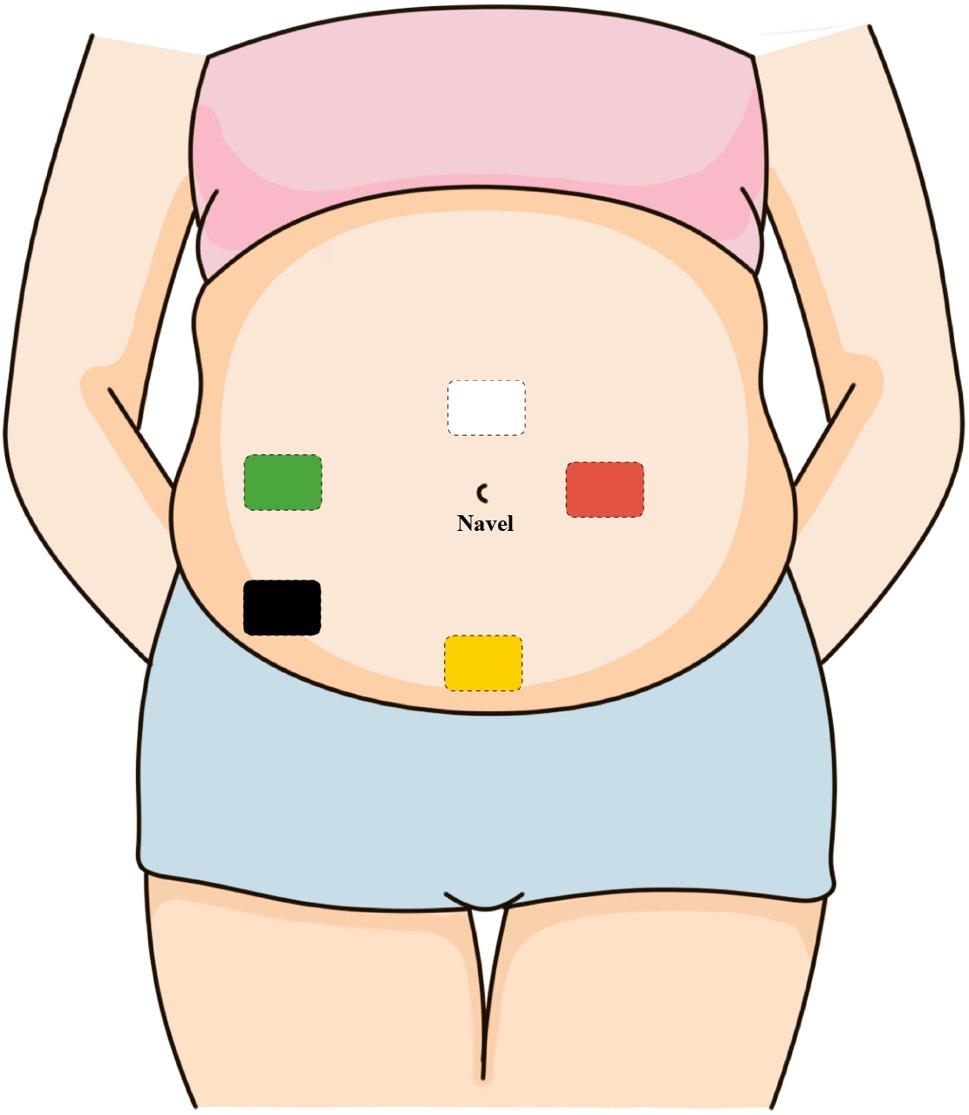
Electrode configuration for uterine electromyography. Note: Blue electrode is 10cm to the right of the center of the navel; white electrode is 3cm directly above the center of the navel; red electrode is 5cm to the left of the center of the navel; yellow electrode is 6cm above the pubic symphysis; black is 10–15cm away from the center of the navel at an angle of 20–45° degrees to the right

The baseline uEMG recording was performed immediately upon admission, prior to initiation of any inpatient therapeutic interventions. Only this pre-treatment uEMG was used in the analyses. The uEMG examination was performed using the Monica AN 24 device (Monica Healthcare Co, UK). Patients were positioned in a resting state, and the abdominal skin was cleaned with a disposable disinfectant solution to remove sweat, oily secretions, or other substances that could interfere with signal acquisition. The electrode placement is shown in Fig. 1. Upon selecting the detected collector, the system confirmed electrode connections and transitioned to the monitoring interface. Following data collection, the uterine contraction curve was exported via the workstation. The curve was generated through analog processing of electrical signals, with signal amplitude peak values ranging from 0 to 100. A peak value ≥ 50 was defined as a valid contraction [12], with a minimum interval of 2 min between consecutive contractions. The following uEMG parameters were calculated:

- Contraction Frequency (contractions/hour) = Number of contractions ÷ Monitoring duration (hours).
- Average Peak Contraction Intensity = Sum of peak signal amplitudes of each contraction ÷ Number of contractions.
- Average Contraction Duration (minutes/contraction) = Sum of the duration of each contraction (minutes) ÷ Number of contractions.

### Clinical data collection and definitions

Clinical data were retrieved from the hospital’s electronic medical records for obstetric inpatients meeting the inclusion and exclusion criteria between January 2018 and May 2025. Collected variables included maternal age, body mass index (BMI), use of ART, gravidity, number of prior deliveries between 12 and 28 weeks, history of intrauterine procedures, congenital uterine anomalies, gestational diabetes, gestational hypertension, reproductive tract infections, and TVCL.

BMI was calculated as pre-pregnancy weight (kg) divided by height (m²), categorized as underweight (< 18.5 kg/m²), normal (18.5–23.9 kg/m²), overweight (24–27.9 kg/m²) or obese (≥ 28 kg/m²). Congenital uterine anomalies included untreated unicornuate uterus, rudimentary horn uterus, bicornuate uterus, arcuate uterus, and complete or incomplete septate uterus. Reproductive tract infections were defined by positive culture results for bacteria, fungi, trichomonas, mycoplasma, or chlamydia in vaginal or cervical secretions. TVCL was measured using transvaginal ultrasound with an empty bladder, placing the probe in the anterior vaginal fornix to measure the linear distance (mm) between the internal and external cervical os in the standard sagittal plane, excluding the funneling portion. Pregnancy outcomes were classified as delivery before 28 weeks or at/after 28 weeks.

### Statistical analysis

#### Comparison of baseline characteristics

Participants were stratified into two groups based on pregnancy outcomes: delivery before 28 weeks and delivery at/after 28 weeks. The distribution of continuous variables was assessed using the Kolmogorov–Smirnov test, complemented by visual inspection of histograms and Q–Q plots. Variables with *P* ≥ 0.05 in the Kolmogorov–Smirnov test or approximately linear Q–Q plots were considered to be approximately normally distributed; otherwise, they were regarded as non-normally distributed. Normally distributed continuous variables were reported as mean ± standard deviation and c e presented as median [Q1, Q3] and compared using the Mann-Whitney U test. Categorical variables were expressed as frequencies (percentages) and compared using the chi-square test or Fisher’s exact test when expected cell counts were < 5.

#### Logistic regression

To evaluate the association between uEMG parameters (contraction frequency, average peak contraction intensity, and average contraction duration) and EPB, three logistic regression models were constructed for each parameter: Model 1 (unadjusted); Model 2 (adjusted for ART and number of previous deliveries between 12 and 28 weeks); and Model 3 (further adjusted for TVCL). Results are reported as odds ratio (OR) with 95% confidence interval (CI). The Youden Index was used to determine optimal cut-off values for each uEMG parameter, which were then dichotomized into binary variables and reintroduced into logistic regression models. A correlation matrix heatmap was created to assess potential collinearity among uEMG parameters.

#### Screening of predictor variables

The whole dataset was used to identify predictor variables associated with EPB. Univariate logistic regression analysis was performed to assess the potential predictive factor of EPB. Factors with a *p*-value < 0.05 in the univariate analysis were included in a multivariate logistic regression analysis, which was developed using stepwise backward elimination. Finally, five variables including ART, TVCL, the number of prior deliveries between 12 and 28 weeks of gestation, contraction frequency, and average contraction duration were included in the final uEMG model.

#### Development of clinical prediction models

To assess the predictive value of uEMG for EPB, two models were constructed: (1) a traditional model incorporating ART, TVCL, and the number of prior deliveries between 12 and 28 weeks; and (2) an enhanced model incorporating these traditional variables plus uEMG parameters (contraction frequency and average contraction duration). Receiver Operating Characteristic (ROC) curves were plotted for both models, and the area under the receiver operating characteristic curve (AUC-ROC) was compared using the DeLong test. The study population was randomly divided into a training set (70%) and an internal validation set (30%). The training set was used to develop the models, and the validation set was used for internal validation. Given that our dataset exhibits class imbalance, with a positive event rate of 20.8%, area under the precision–recall curve (AUC-PR) was employed to further evaluate the model’s performance, as it is more sensitive to the identification of rare positive cases. A nomogram for risk scoring was developed based on the training set. The optimal cut-off value, determined using the Youden Index, was used to stratify patients into low-risk and high-risk subgroups. The chi-square test was used to compare the actual EPB rates between these subgroups in both the training and validation sets.

Model calibration was evaluated by plotting calibration curves and using bootstrap resampling with 1,000 repetitions in the training and set; apparent and bias-corrected curves were displayed for both the training and validation sets. Decision curve analysis (DCA) was performed to quantify the net clinical benefit of the models across a range of threshold probabilities. To further examine model robustness and potential overfitting, we conducted five-fold cross-validation, reporting the mean AUC-ROC and accuracy across folds.

## Result

**Fig. 2:**
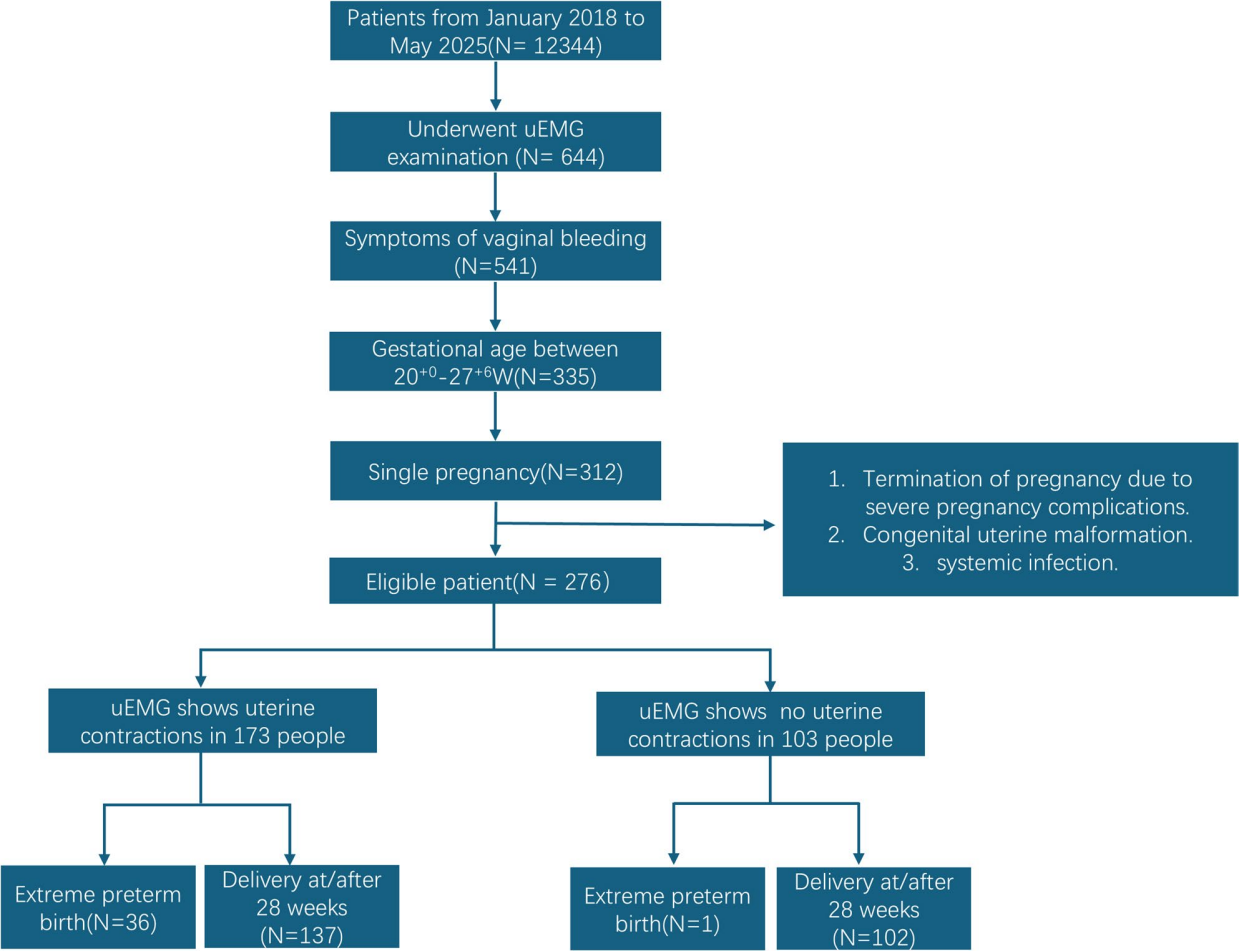
Flow chart of the selection of the study population. Note: uEMG: uterine electromyography

UEMG monitoring detected no uterine contraction signals in 103 cases, of which 102 delivered at/after 28 weeks and 1 delivered before 28 weeks. UEMG monitoring detected uterine contraction signals in 173 cases, of which 137 delivered at/after 28 weeks and 36 delivered before 28 weeks. After excluding the 103 patients without contraction signals, 173 patients were included in the final analysis. The flowchart of participants through the study is shown in Fig. 2. Supplementary Table 1 presents the baseline characteristics of the no-contraction and contraction groups, revealing significant differences in age, BMI, and TVCL (*P* < 0.05), with no significant differences in other baseline parameters.

This study ultimately included 173 patients. Baseline characteristics of the non-EBP group (delivery at or after 28 weeks, *N* = 137) and the EBP group (*N* = 36) were compared, as shown in Table 1. The EBP group exhibited significantly higher contraction frequency, intensity, and duration compared to the non-EBP group. Additionally, significant differences were observed in the use of ART, the number of prior deliveries between 12 and 28 weeks of gestation, and TVCL (*P* < 0.05), while other baseline characteristics showed no significant differences.

**Table 1:** Baseline characteristics

	Delivery after 28 weeks (*N* = 137)	Delivery before 28 weeks (*N* = 36)	*P*
Age^a^	32.30 ± 4.33	33.25 ± 3.74	0.230
BMI (kg/m2)^c^			0.128
Slim	19 (13.9)	1 (2.8)	
Normal	98 (71.5)	27 (75.0)	
Overweight/Obese	20 (14.6)	8 (22.2)	
ART^c^	34 (24.8)	19 (52.8)	**0.002**
Gravidity^b^	3.00 [2.00, 5.00]	3.00 [2.00, 4.00]	0.095
Number of previous deliveries between 12-28weeks^b^	0.00 [0.00, 1.00]	0.00 [0.00, 1.00]	**0.029**
Intrauterine procedures^c^	91 (66.4)	18 (50.0)	0.105
Gestational diabetes^c^	30(21.8)	7 (19.4)	0.815
Gestational hypertension^d^	9 (6.6)	2 (5.6)	1
Reproductive tract infections^c^	17 (12.4)	7 (19.4)	0.415
TVCL (mm)^b^	21.00 [9.00, 32.00]	0.00 [0.00, 21.25]	**< 0.001**
Contraction frequency^b^ (contractions/hour)	1.71 [0.95, 3.00]	3.23 [2.16, 5.03]	**< 0.001**
Average peak contraction intensity^b^	62.50 [54.52, 72.29]	72.15 [66.93, 86.28]	**< 0.001**
Average contraction duration^b^(minutes/contraction)	1.34 [1.21, 1.56]	1.73 [1.58, 2.07]	**< 0.001**

Data are presented as mean ± SD, median [IQR], or n (%)

*BMI* body mass index, *ART* assisted reproductive technology, *TVCL* transvaginal cervical length

^a^ Student’s t test is used to analyze the differences among groups. P value < 0.05 was considered statistically significant

^b^ Mann–Whitney U test is used to analyze the differences among groups. *P* value < 0.05 was considered statistically significant

^c^ Chi-square test is used to analyze the differences among groups. *P* value < 0.05 was considered statistically significant

^d^ Fisher’s exact test is used to analyze the differences among groups. *P* value < 0.05 was considered statistically significant

The correlation heatmap matrix (Supplemental Fig. 1) illustrates the relationships among uEMG parameters. Average peak contraction intensity and average contraction duration showed a strong positive correlation (*r* = 0.61[0.50,0.69], *P* < 0.05). Contraction frequency exhibited a moderate positive correlation with average peak contraction intensity (*r* = 0.36[0.23,0.49], *P* < 0.05) and a weaker correlation with average contraction duration (*r* = 0.27[0.13,0.41], *P* < 0.05).

As shown in Table 2, all three uEMG parameters were significantly associated with an increased risk of EPB in the unadjusted model (Model 1). These associations remained statistically significant after adjusting for covariates in Model 2 (ART and number of previous deliveries between 12 and 28 weeks) and Model 3 (further adjusted for TVCL). When uEMG parameters were dichotomized using optimal cut-off values determined by the Youden Index, patients in the higher-risk group had a significantly increased risk of EPB compared to the reference group after adjusting for all covariates (*P* < 0.05).

**Table 2:** Association of uEMG related parameters with EPB

	Model 1^a^		Model 2 ^b^		Model 3 ^c^	
	OR (95% CI)	*P* value	OR (95% CI)	*P* value	OR (95% CI)	*P* value
Contraction Frequency(continous)	1.21(1.07,1.40)	**0.004**	1.21(1.07, 1.41)	**0.007**	1.23(1.07,1.45)	**0.008**
Groups						
Q1(≤ 2.06)	Ref.		Ref.		Ref.	
Q2(> 2.06)	5.06(2.24,12.65)	**< 0.001**	5.55(2.39,14.31)	**< 0.001**	4.97(2.01,13.57)	**< 0.001**
Average Peak Contraction Intensity(continous)	1.05(1.02,1.08)	**< 0.001**	1.05(1.02,1.08)	**< 0.001**	1.04(1.01,1.08)	**0.004**
Groups						
Q1(≤ 66.7)	Ref.		Ref.		Ref.	
Q2(> 66.7)	10.60(4.60,27.00)	**< 0.001**	10.82(4.58,28.28)	**< 0.001**	14.67(5.38,47.17)	**< 0.001**
Average Contraction Duration(continous)	15.17(5.39,48.29)	**< 0.001**	15.29(5.30,49.43)	**< 0.001**	17.84(5.34,69.86)	**< 0.001**
Groups						
Q1(≤ 1.56)	Ref.		Ref.		Ref.	
Q2(> 1.56)	5.38(2.38,13.45)	**< 0.001**	5.18(2.24,13.21)	**< 0.001**	4.22(1.74,11.18)	**0.002**

*uEMG *Uterine electromyography, *EPB* Extremely preterm birth, *OR* odds ratio, *CI* confidence interval, *Ref* reference

Model 1: Unadjusted for nothing

Model 2: Adjusted for ART and number of previous deliveries between 12-28 weeks

Model 3: Model 2 further adjusted for TVCL

**Fig. 3:**
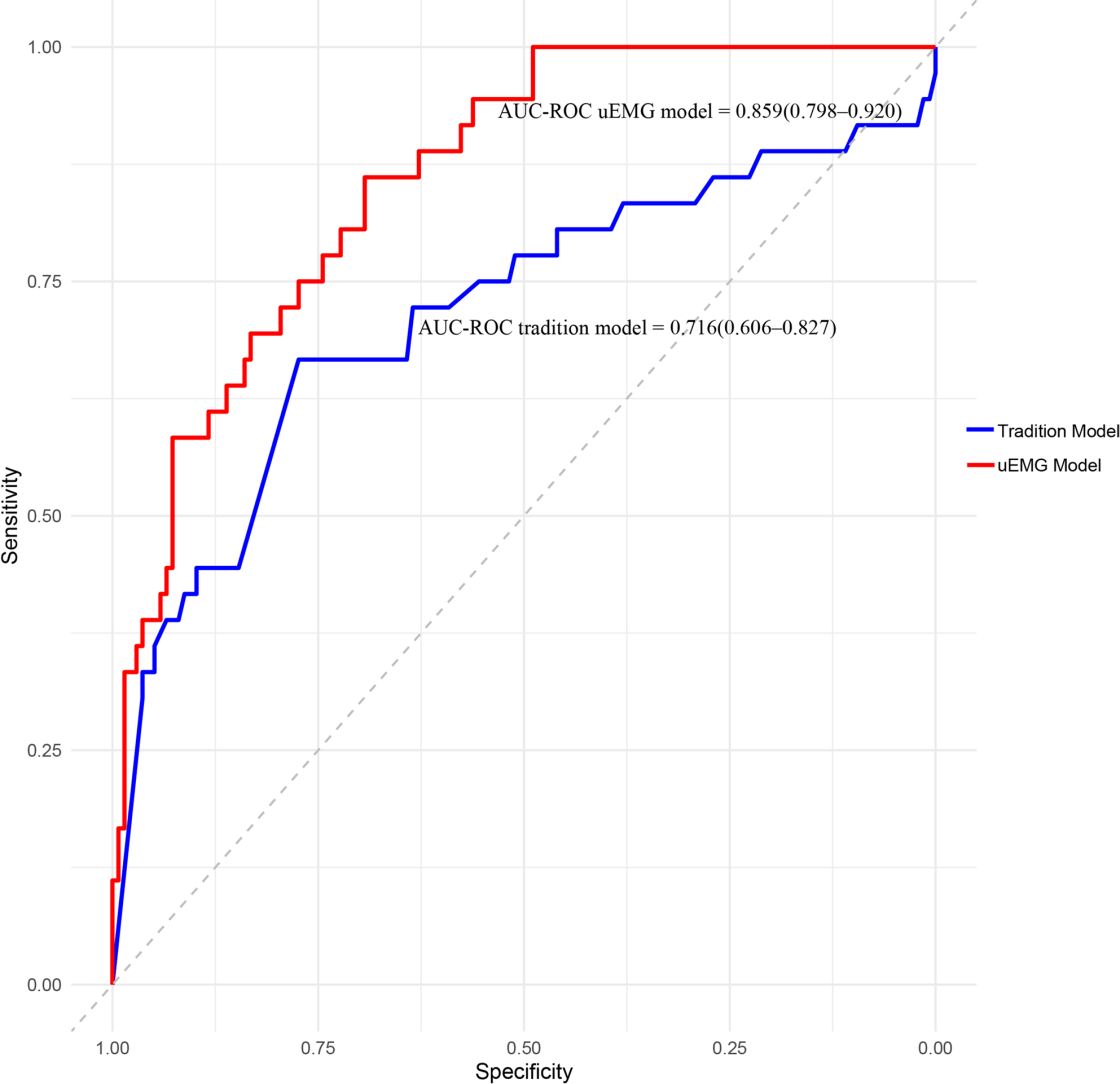
The AUC-ROC of uEMG model and traditional model. Note: uEMG: uterine electromyography; AUC-ROC: The area under the receiver operating characteristic curve; ART: assisted reproductive technology; TVCL: transvaginal cervical length. Traditional model predictive factors: ART, TVCL, and the number of prior deliveries between 12 and 28 weeks. UEMG Model predictive factors: ART, TVCL, the number of prior deliveries between 12 and 28 weeks, contraction frequency and average contraction duration

Two predictive models were developed based on the above findings and backward elimination. The traditional model included ART, TVCL, and the number of prior deliveries between 12 and 28 weeks. The uEMG model incorporated these traditional predictors plus uEMG parameters (contraction frequency and average contraction duration). ROC curves for both models are shown in Fig. 3. The AUC-ROC for the traditional model was 0.716 (95% CI: 0.606–0.827), while the uEMG model achieved an AUC-ROC of 0.859 (95% CI: 0.798–0.920). The difference in AUC-ROC between the two models was statistically significant (*P* < 0.05, DeLong test).

The uEMG model was selected as the final predictive model, and the dataset was split into a training set (70%, *n* = 121) and an internal validation set (30%, *n* = 52). Supplementary Table 2 compares the baseline characteristics of the training and validation sets, showing no significant differences. ROC curves for the training and validation sets are presented in Supplemental Fig. 2. The AUC-ROC for the training set was 0.812 (95%CI:0.731–0.894), and for the validation set was 0.915(95%CI:0.895-1). PR curves for the training and validation sets are presented in Supplemental Fig. 3, The AUC-PR for the training set was 0.548 (95% CI: 0.395–0.746), and for the validation set was 0.877(95%CI:0.683–0.987).

**Fig. 4:**
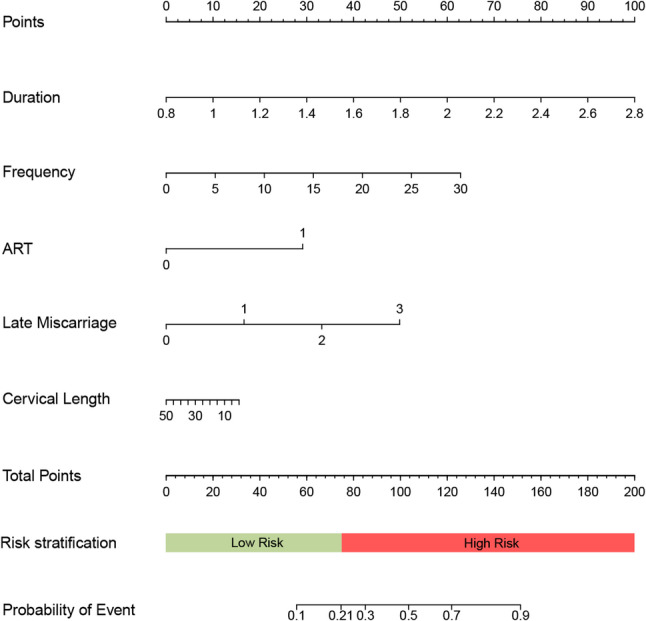
Nomogram for predicting EPB. Note: EPB: Extremely Preterm Birth; ART: assisted reproductive technology

To evaluate potential overfitting, five-fold cross-validation was performed (Table 3). The mean AUC-ROC was 0.823(0.735–0.889), with accuracy ranging from 0.743 to 0.886 (mean = 0.815), indicating robust model performance. A nomogram was developed to facilitate clinical application (Fig. 4). In the training set, the optimal probability cut-off value, determined by the Youden Index, was 0.193 (AUC-ROC = 0.812, 95% CI: 0.731–0.895), corresponding to a nomogram score of 76. Patients scoring above 76 were classified as high-risk for EPB, while those scoring below 76 were considered low-risk for EPB. For example, Patient A, with no prior deliveries between 12 and 28 weeks, natural conception, a TVCL of 10 mm, a contraction frequency of 5 times/hour, and an average contraction duration of 2 min, had a nomogram score of approximately 85, indicating high risk for EPB. Her pregnancy outcome was EPB, consistent with the nomogram’s prediction.

**Table 3:** Fivefold cross-validation of final predictive model

	AUC-ROC	*R* ^2^	Accuracy
Fold1	0.889	0.297	0.853
Fold2	0.867	0.312	0.771
Fold3	0.806	0.330	0.743
Fold4	0.816	0.304	0.886
Fold5	0.735	0.340	0.824
Average	0.823	0.317	0.815

*AUC-ROC* Area Under the Receiver Operating Characteristic Curve, *R*^2^ Coefficient of Determination

**Fig. 5:**
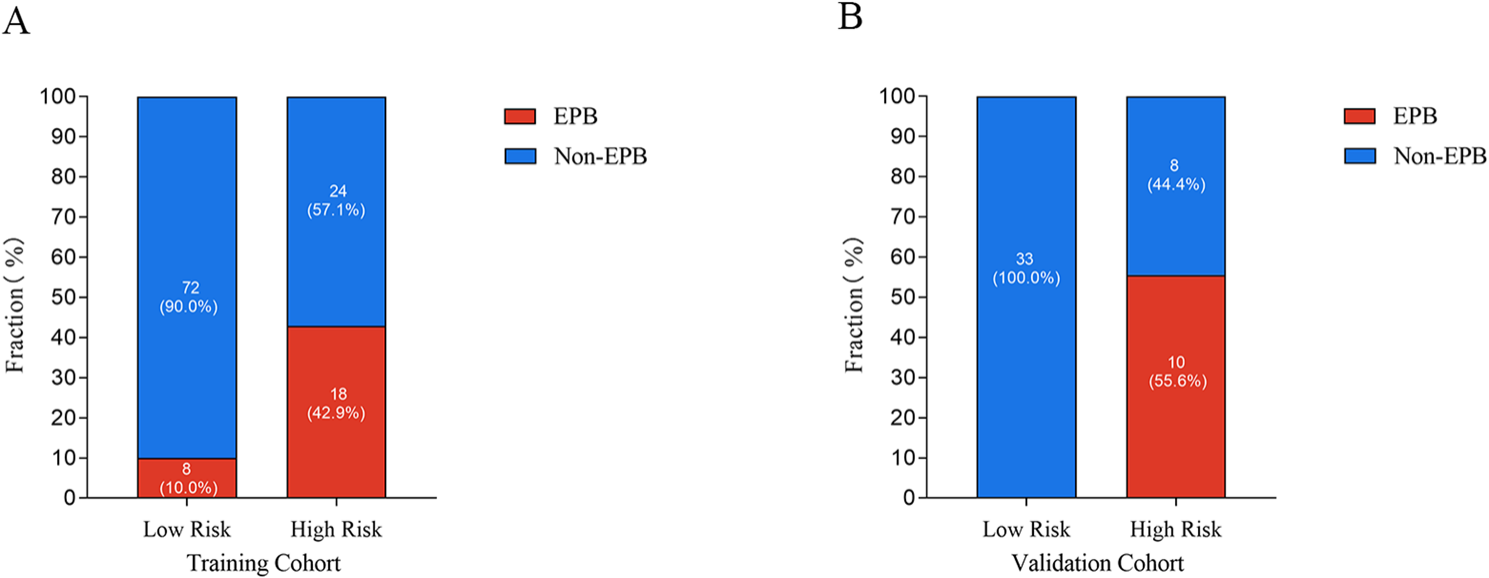
EPB in training cohort (**A**), and validation cohort (**B**). Note: EPB: Extremely Preterm Birth. Patients were categorized into two high-risk and low-risk based on the optimal cut-off value of 76 points, as determined using the Youden index, for the nomogram risk scores

In the training set, the predicted EPB rate was 9.4% for the low-risk group (actual rate: 10.0%) and 44.0% for the high-risk group (actual rate: 42.9%), with significant differences between groups (*P* < 0.001). In the validation set, the predicted EPB rate was 8.5% for the low-risk group (actual rate: 0%) and 45.0% for the high-risk group (actual rate: 55.6%), with significant differences (*P* < 0.001). These findings, illustrated in Fig. 5, demonstrate the nomogram’s effectiveness in stratifying patients and predicting EPB risk.

Calibration analysis revealed good performance in the training set, with the bias-corrected curve indicating slight overfitting. In the validation set, calibration was less robust, with the apparent curve deviating from the ideal line, with a tendency toward risk overestimation at higher predicted probabilities, consistent with small-sample instability and potential overfitting. (Supplemental Fig. 4).

DCA showed a high net benefit at low probability thresholds (0.0–0.1) in the training cohort. At higher thresholds (> 0.6), the net benefit approached 0, indicating reduced clinical utility. In the validation cohort, the net benefit was generally higher than in the training set (Supplemental Fig. 5).

## Discussion

In this single-center retrospective study, we evaluated whether uEMG-derived parameters provide additional predictive value for EPB. Our findings indicate that elevated contraction frequency and average contraction duration, along with a history of ART, prior deliveries between 12 and 28 weeks of gestation, and shortened TVCL, are independent predictors of EPB. Compared to conventional clinical features evaluation systems, The multifactorial predictive model developed in this study integrates bioelectrical signals (uEMG parameters) with clinical features (ART, TVCL, obstetric history), outperforming traditional models with higher AUC-PR and AUC-ROC, providing a quantitative tool for risk stratification.

Earlier studies have demonstrated that uEMG characteristics can distinguish women who will deliver preterm before 37 weeks from those who will deliver at term [16–18], and that uEMG could outperform tocodynamometry and some conventional clinical markers in this context [19, 20]. However, most of these studies focused on overall preterm birth, whereas EPB represents a smaller but clinically distinct subgroup with disproportionately high neonatal morbidity and mortality. By restricting the outcome to EPB, our study addresses a more severe and clinically critical endpoint. The observation that uEMG parameters retain predictive value even after accounting for ART, prior very preterm delivery, and TVCL suggests that electrical signatures of uterine activity capture a dimension of risk that is not fully reflected by static anatomical or historical factors.

The clinical implications of these findings are significant. In clinical practice, uEMG can serve as a valuable complementary tool to traditional assessment methods, like TVCL and obstetric history, which are recognized as robust predictors of preterm birth and are integrated into clinical guidelines [21–24].This study further found that the average contraction duration, contractions frequency and average peak contraction intensity are significantly elevated in EPB group. Prolonged contraction duration and contractions frequency may reflect dysregulated calcium ion channel activity in the uterine myometrium, leading to disrupted contraction rhythms and accumulated mechanical stress [25]. Notably, although average peak contraction intensity was positively associated with EPB in univariate analysis, this correlation was not sustained in the multivariate model. Previous evidence indicates that cervical effacement and dilatation depend more on the overall uterine activity accumulated over time than on the peak strength of individual contractions [26]. In clinical practice, this overall activity is often quantified in Montevideo units, which integrate contraction intensity, duration, and frequency over a given time interval [27], and better reflect the effectiveness of uterine contractions in driving cervical change than isolated peak amplitudes. This may also explain why, in our study, contraction frequency and duration emerged as stronger predictors of EPB than the peak intensity.

Additionally, our findings reconfirm ART as a risk factor for EPB, consistent with previous research [28, 29]. The etiology of ART-related preterm birth is multifactorial, including the technology itself, premature rupture of membranes, uterine factors, placenta factor and underlying female infertility etiology, all of which may contribute to preterm delivery [30–34].

During internal validation, the model demonstrated higher performance in both AUC-PR and AUC-ROC on the validation set compared to the training set. This phenomenon is probably related to the limited sample size. In particular, the small number of EPB events in the validation set. With only 52 women and 10 cases of events, the AUC-ROC estimate is statistically unstable and more sensitive to the specific random split of the data. Furthermore, calibration curve revealed suboptimal performance in the validation set, with the calibration curve deviating from the ideal line. This suggests potential overfitting in the training set or differences in data distribution between the training and validation sets. These findings underscore the need for further refinement and validation of the model.

### Strengths and limitations

This study has several strengths. First, it introduces contraction-related uEMG parameters as independent predictors of EPB, providing a novel approach to assess EPB risk. Second, the use of uEMG for monitoring uterine contractions offers a non-invasive, dynamic, and intuitive tool for clinical practice. Finally, our findings support the expanded clinical application of uEMG and enhance its reliability as a predictive tool for EPB, laying the groundwork for future research and implementation.

However, this study also has limitations. First, our cohort consisted exclusively of hospitalized women with EPB risk at a tertiary referral center who were selected for uEMG monitoring based on clinical suspicion. What’s more, women without detectable uterine contractions on uEMG were not included in model development. Consequently, the observed EPB rate is much higher than in the general obstetric population. This reflects the underlying high-risk case mix and implies that our model is currently applicable to a similar high-risk population, rather than to unselected pregnant women. Future studies including lower-risk or population without measurable contractions are needed to develop a more robust predictive model. Second, the numerically higher AUC in the small internal validation subset, together with imperfect calibration and the limited number of events, suggests that our model is at risk of overfitting and that its apparent performance is likely optimistic. The five-fold cross-validation results indicate that true discrimination is more modest, but these internal validation procedures cannot replace external validation. Larger multicenter prospective studies are required to assess the stability, calibration, and generalizability of the model across different clinical settings and patient populations before it can be considered for routine clinical application. Finally, post-uEMG interventions (e.g., tocolytics, corticosteroids) may have influenced delivery timing, potentially attenuating or modifying associations between baseline risk and outcomes. With the limited number of events, we did not model multiple time-varying treatments to avoid overfitting and collider bias; future prospective studies with standardized treatment capture are needed to disentangle prognostic information from treatment effects.

## Conclusions

In conclusion, this study confirms that uEMG parameters, specifically contraction frequency and average contraction duration, are independent predictors of EPB. The predictive model, integrating these uEMG parameters with clinical indicators such as ART history, prior deliveries between 12 and 28 weeks, and TVCL, demonstrates better discrimination than a traditional clinical model alone and may support more refined risk stratification and individualized management. As a non-invasive and dynamic monitoring method, uEMG has the potential to complement established assessment tools in clinical practice. However, our findings are derived from a single-center retrospective cohort with a relatively limited sample size, and women without detectable contractions were not included in model development. Given the statistical instability observed in internal validation and the calibration discrepancies between training and validation sets, the present model should be regarded as exploratory and internally validated only. The present results should therefore be interpreted as exploratory risk-stratification tool for high-risk women, whether its use to guide clinical management can improve outcomes requires prospective evaluation.

## Supplementary Information

Supplementary Material 1.

Supplementary Material 2.

Supplementary Material 3.

Supplementary Material 4.

Supplementary Material 5.

Supplementary Material 6.

Supplementary Material 7.

## Data Availability

The data that supports the findings of this study are available from the corresponding author upon reasonable request.

## Abbreviations

EPB: Extremely preterm birth
uEMG: Uterine electromyography
ART: Assisted reproductive technology
TVCL: Transvaginal cervical length measurement
BMI: Body mass index
ROC: Receiver Operating Characteristic
AUC-ROC: Area Under the Receiver Operating Characteristic Curve
AUC-PR: Area Under the Precision Recall Curve
