# Supplementary figures and images for: Uterine electromyography as a new predictor of extremely preterm birth: a multifactorial model integrating clinical and bioelectrical parameters
Source: BMC Pregnancy Childbirth. 2025 Dec 26;26:99. doi: 10.1186/s12884-025-08539-3 (PMC12849207; doi:10.1186/s12884-025-08539-3)

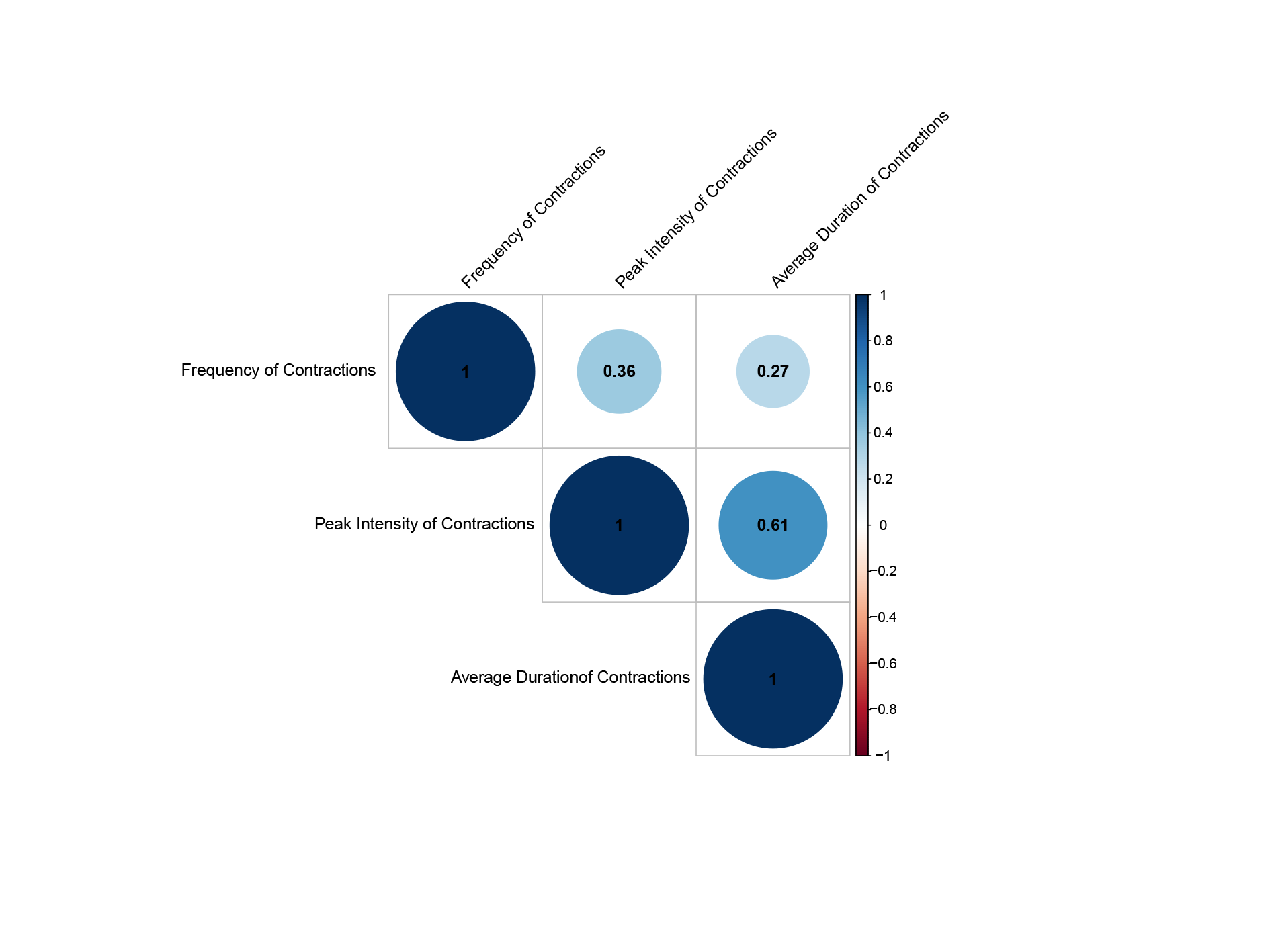

Supplement: Supplementary file 1 — Supplementary Material 1. [file 12884_2025_8539_MOESM1_ESM.tif]

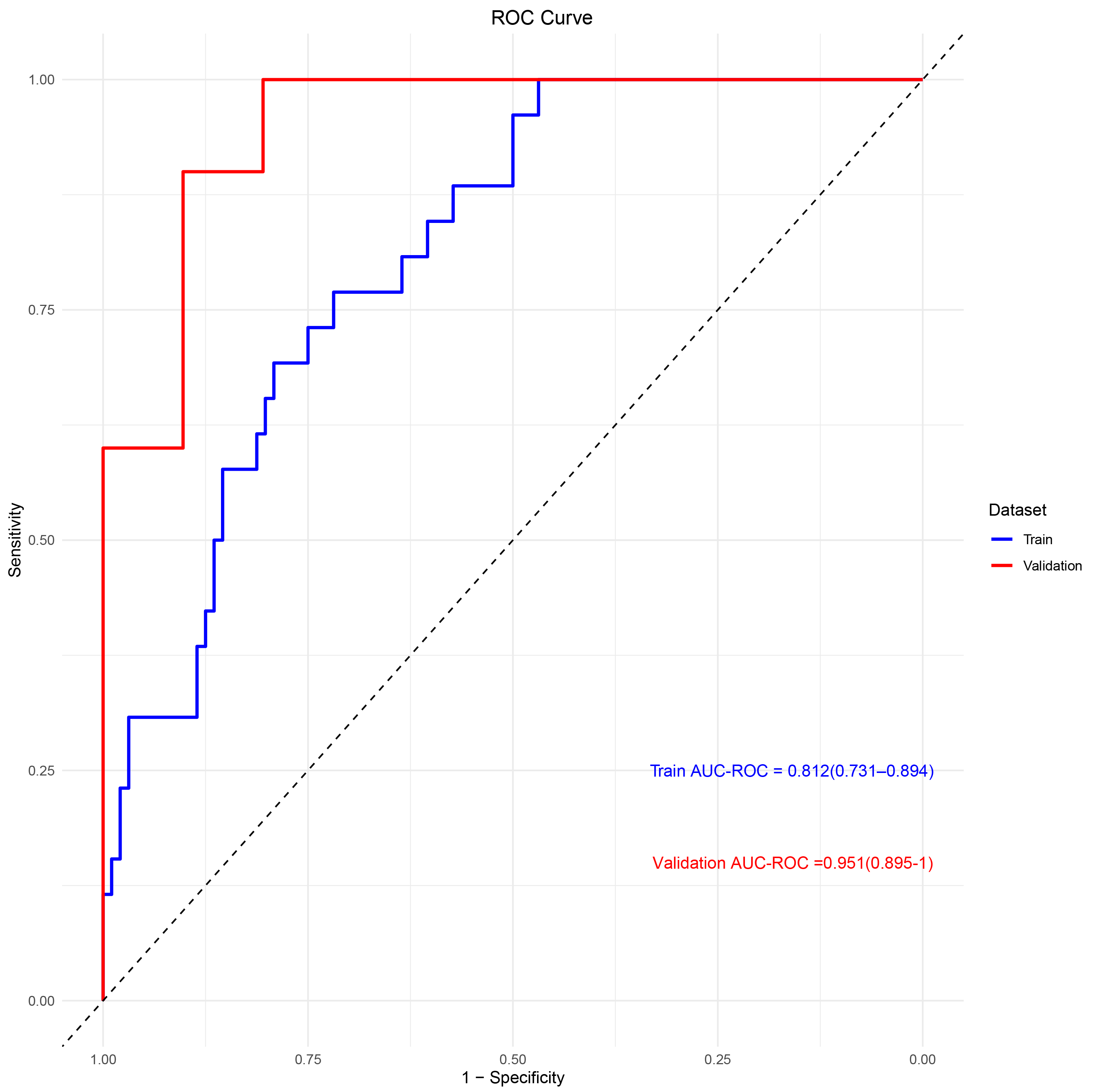

Supplement: Supplementary file 2 — Supplementary Material 2. [file 12884_2025_8539_MOESM2_ESM.tif]

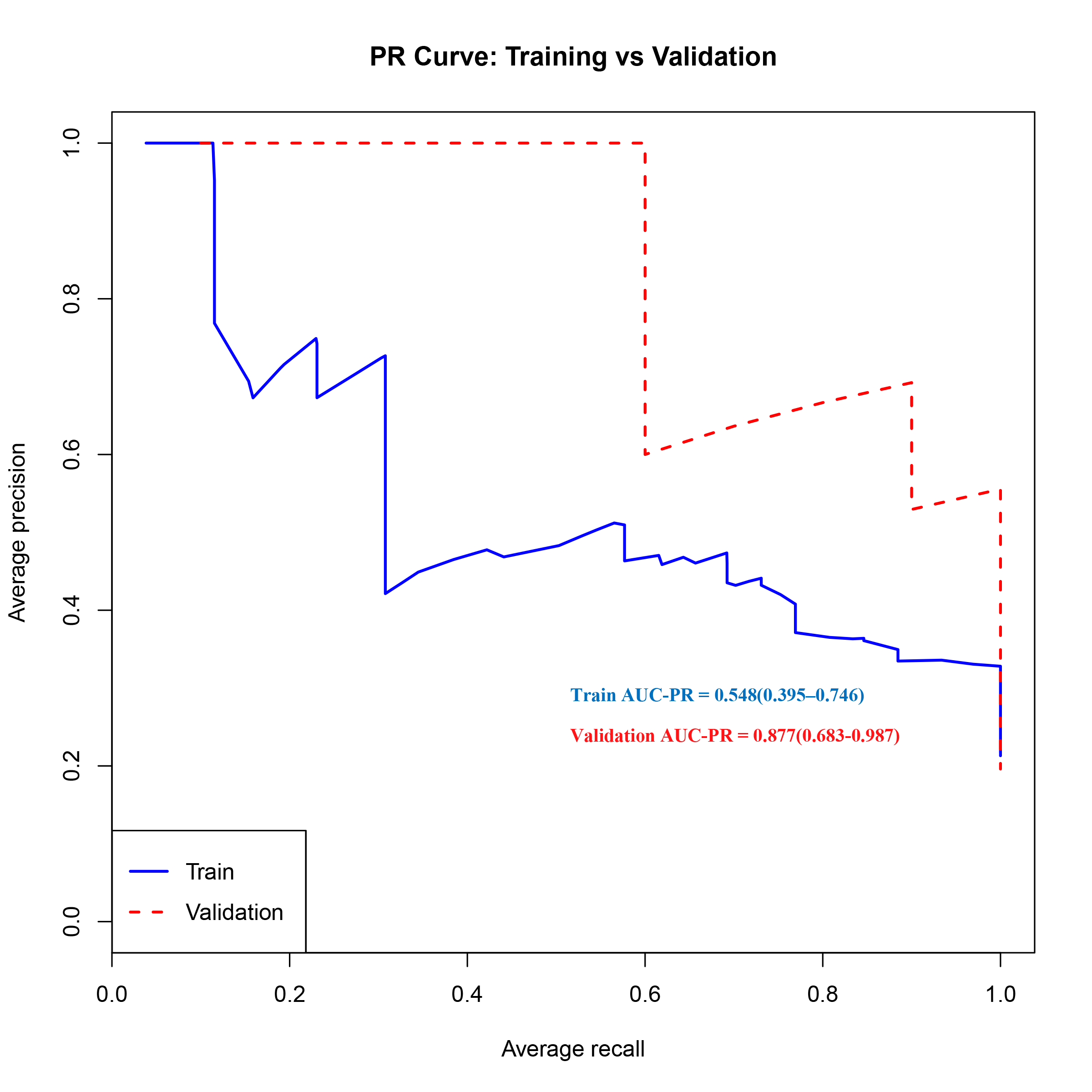

Supplement: Supplementary file 3 — Supplementary Material 3. [file 12884_2025_8539_MOESM3_ESM.tif]

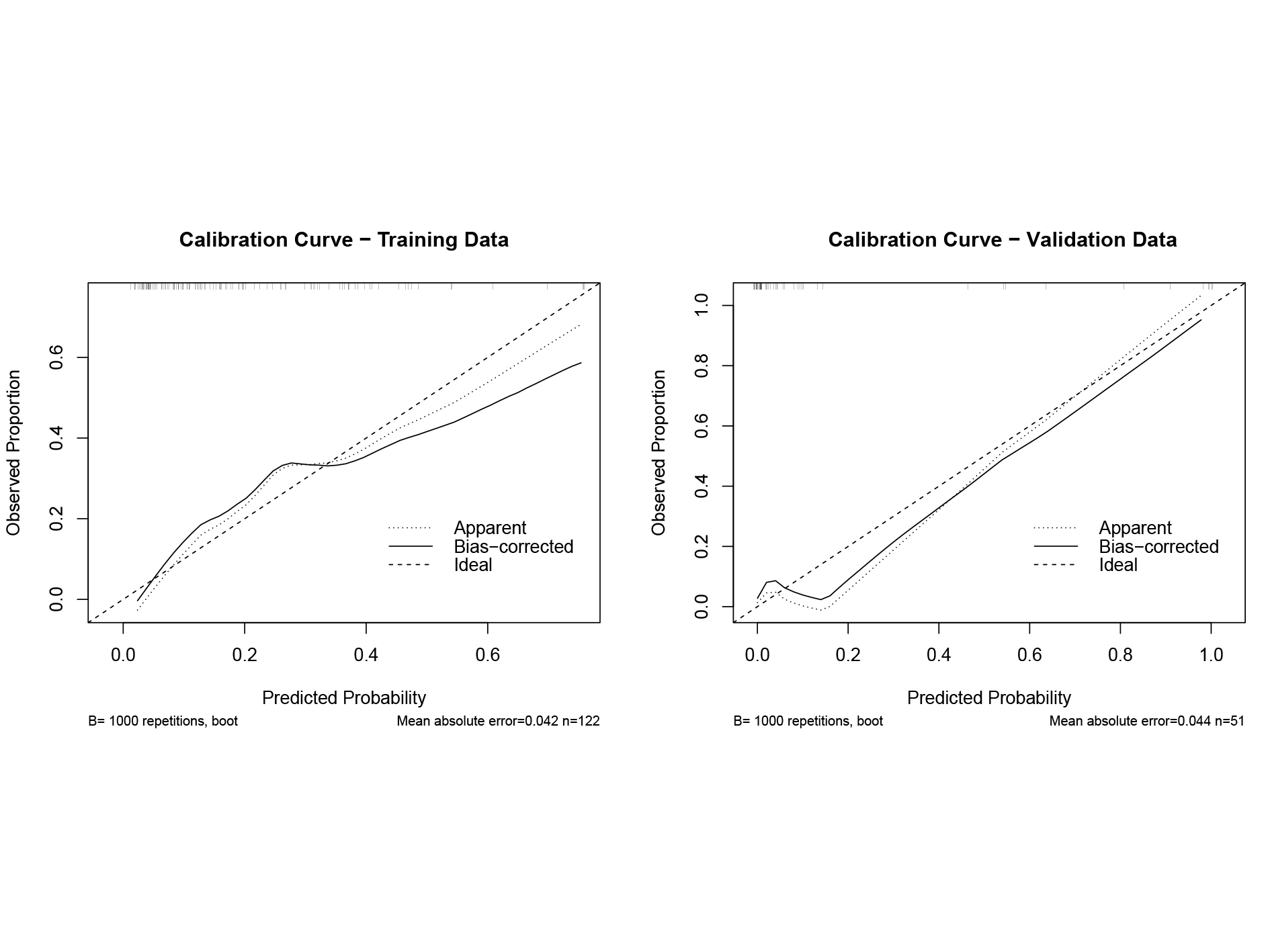

Supplement: Supplementary file 4 — Supplementary Material 4. [file 12884_2025_8539_MOESM4_ESM.tif]

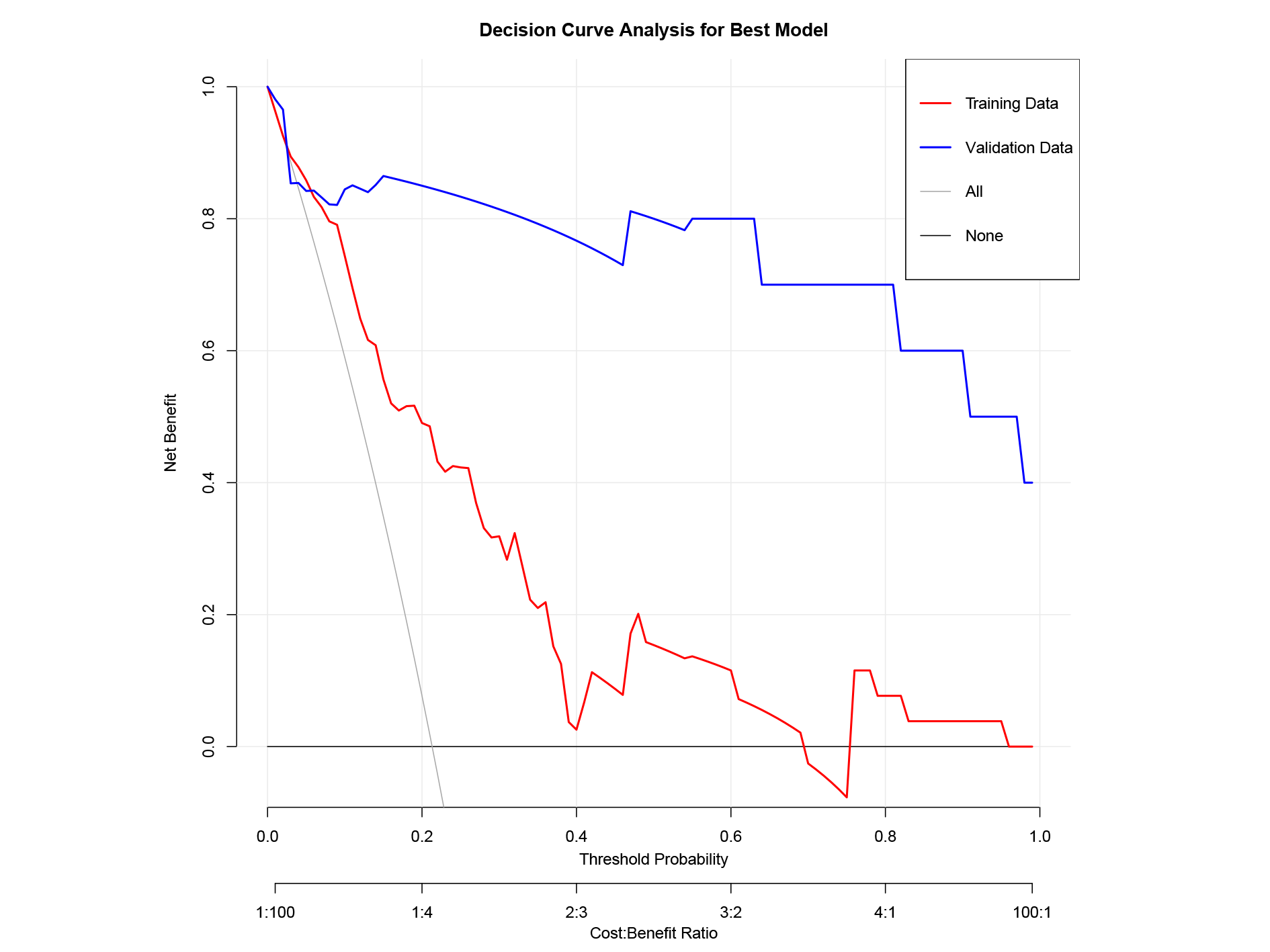

Supplement: Supplementary file 5 — Supplementary Material 5. [file 12884_2025_8539_MOESM5_ESM.tif]
