# Supplementary material for: Uterine electromyography as a new predictor of extremely preterm birth: a multifactorial model integrating clinical and bioelectrical parameters
Source: BMC Pregnancy Childbirth. 2025 Dec 26;26:99. doi: 10.1186/s12884-025-08539-3 (PMC12849207; doi:10.1186/s12884-025-08539-3)
Supplement: Supplementary file 6 — Supplementary Material 6. [file 12884_2025_8539_MOESM6_ESM.docx]

Supplemental Table1: Baseline Characteristics of Uterine-Contraction Group and No-Uterine-Contraction Group

|  | No Uterine Contraction Group  （N = 103） | Uterine Contraction Group  （N = 173） | *P* |
| --- | --- | --- | --- |
| Age^a^ | 34.05±4.17 | 32.50±4.22 | **0.003** |
| BMI^c^（kg/m2） |  |  | **<0.001** |
| Slim | 1 (1.0) | 20 (11.6) |  |
| Normal | 95 (92.2) | 125 (72.3) |  |
| Overweight/ Obese | 7 (6.8) | 28 (16.2) |  |
| ART^c^ | 39 (37.9) | 53 (30.6) | 0.271 |
| Gravidity^b^ | 3.00 [3.00, 5.00] | 3.00 [2.00, 4.00] | 0.138 |
| Number of previous deliveries between 12-28weeks^b^ | 0.00 [0.00, 1.00] | 0.00 [0.00, 1.00] | 0.609 |
| Intrauterine procedures^c^ | 71 (68.9) | 109 (63.0) | 0.385 |
| Gestational diabetes^c^ | 19 (18.4) | 37(21.4) | 0.665 |
| Gestational hypertension^d^ | 2 (1.9) | 11 (6.4) | 0.167 |
| Reproductive Tract Infections^c^ | 17 (16.5) | 24 (13.9) | 0.675 |
| TVCL^b^（mm） | 21.00 [16.00, 30.00] | 18.00 [7.00, 32.00] | **0.015** |
| Gestational Age at Delivery^a^(days) | 231.3±42.6 | 260.4±18.8 | **<0.001** |

Data are presented as mean ± SD, median [IQR], or n (%).

BMI: body mass index; ART: assisted reproductive technology; TVCL: transvaginal cervical length.

^a^ Student’s t test is used to analyze the differences among groups. P value < 0.05 was considered statistically significant

^b^ Mann–Whitney U test is used to analyze the differences among groups. P value < 0.05 was considered statistically significant

^c^ Chi-square test is used to analyze the differences among groups. P value < 0.05 was considered statistically significant

^d^ Fisher’s exact test is used to analyze the differences among groups. P value < 0.05 was considered statistically significant

Supplemental Table2: Characteristics of test set and training set

| **Characteristic** | **Test set**  N = 51 | **Training set**  N = 122 | ***P*** |
| --- | --- | --- | --- |
| Contraction Frequency^a^ | 1.41[1.23, 1.76] | 1.45[1.25, 1.64)] | 0.948 |
| Average Contraction Duration^a^ | 1.89[1.00, 3.87) | 2.24[1.00, 3.87) | 0.499 |
| ART^b^ | 18 (35.3%) | 35 (28.7%) | 0.497 |
| Number of previous deliveries between 12-28weeks^a^ | 0.00 [0.00, 1.00] | 0.00 [0.00, 1.00] | 0.403 |
| TVCL^a^（mm） | 21[6, 30] | 17[7, 34] | 0.916 |
| Delivery before 28 weeks of gestation ^b^ | 10(19.6%) | 26 (21.3%) | 0.963 |

Data are presented as mean ± SD, median [IQR], or n (%).

ART：assisted reproductive technology; TVCL: transvaginal cervical length

^a^ Mann–Whitney U test is used to analyze the differences among groups. *P* value < 0.05 was considered statistically significant

^b^ Chi-square test is used to analyze the differences among groups. *P* value < 0.05 was considered statistically significant
