# Supplementary material for: Uterine electromyography as a new predictor of extremely preterm birth: a multifactorial model integrating clinical and bioelectrical parameters
Source: BMC Pregnancy Childbirth. 2025 Dec 26;26:99. doi: 10.1186/s12884-025-08539-3 (PMC12849207; doi:10.1186/s12884-025-08539-3)
Supplement: Supplementary file 7 — Supplementary Material 7. [file 12884_2025_8539_MOESM7_ESM.docx]

**Supplemental Figure 1 Correlation Matrix Heatmap**

Correlation Matrix for Frequency of Contractions, Peak Intensity of Contractions, Average Duration of Contractions

**Supplemental Figure 2 The AUC-ROC for the prediction of EPB within the training and validation cohorts.**

Note: AUC-ROC: The area under the receiver operating characteristic curve; EPB: Extremely Preterm Birth; ROC: Receiver Operating Characteristic

The ROC curves, depicted by red and bule lines, illustrate the model’s performance at various thresholds for the training (blue) and validation (red) cohorts.

**Supplemental Figure 3 AUC-PR for the prediction of EPB within the training and validation cohorts.**

Note: AUC-PR: The area under the precision recall curve; EPB: Extremely Preterm Birth; PR: precision recall

**Supplemental Figure 4 Calibration curves for the prediction of EPB within the training and validation cohorts.**

Note: EPB: Extremely Preterm Birth

**Supplemental Figure5 DCA for the prediction of EPB within the training and validation cohorts.**

Note: DCA: Decision Calibration analysis; EPB: Extremely Preterm Birth
